# Supplementary material for: Minimizing IP issues associated with gene constructs encoding the Bt toxin - a case study
Source: BMC Biotechnol. 2024 Jun 3;24:37. doi: 10.1186/s12896-024-00864-3 (PMC11145813; doi:10.1186/s12896-024-00864-3)
Supplement: Supplementary file 3 — Supplementary Material 3 [file 12896_2024_864_MOESM3_ESM.docx]

Supplementary Table 2. Comparison of codon use frequency between the original *Cry1C* and *Cry1C^M^* genes.

| **Amino Acid** | **Codon** | **Number** | | **Fraction** | |
| --- | --- | --- | --- | --- | --- |
|  |  | ***Cry1C*** | ***Cry1C^M^*** | ***Cry1C*** | ***Cry1C^M^*** |
| Ala | GCG | 0 | 5 | 0.00 | 0.16 |
| Ala | GCA | 0 | 9 | 0.00 | 0.29 |
| Ala | GCT | 31 | 11 | 1.00 | 0.35 |
| Ala | GCC | 0 | 6 | 0.00 | 0.19 |
| Cys | TGT | 4 | 3 | 1.00 | 0.75 |
| Cys | TGC | 0 | 1 | 0.00 | 0.25 |
| Asp | GAT | 27 | 16 | 0.93 | 0.55 |
| Asp | GAC | 2 | 13 | 0.07 | 0.45 |
| Glu | GAG | 36 | 21 | 0.93 | 0.57 |
| Glu | GAA | 1 | 16 | 0.03 | 0.43 |
| Phe | TTT | 0 | 11 | 0.00 | 0.28 |
| Phe | TTC | 40 | 29 | 1.00 | 0.72 |
| Gly | GGG | 0 | 11 | 0.00 | 0.23 |
| Gly | GGA | 48 | 15 | 1.00 | 0.31 |
| Gly | GGT | 0 | 10 | 0.00 | 0.21 |
| Gly | GGC | 0 | 12 | 0.00 | 0.25 |
| His | CAT | 8 | 5 | 1.00 | 0.63 |
| His | CAC | 0 | 3 | 0.00 | 0.37 |
| Ile | ATA | 0 | 12 | 0.00 | 0.28 |
| Ile | ATT | 6 | 17 | 0.13 | 0.35 |
| Ile | ATC | 42 | 19 | 0.88 | 0.40 |
| Lys | AAG | 4 | 2 | 0.80 | 0.40 |
| Lys | AAA | 1 | 3 | 0.20 | 0.60 |
| Leu | TTG | 0 | 10 | 0.00 | 0.18 |
| Leu | TTA | 1 | 5 | 0.02 | 0.09 |
| Leu | CTG | 0 | 9 | 0.00 | 0.16 |
| Leu | CTA | 0 | 7 | 0.00 | 0.12 |
| Leu | CTT | 56 | 14 | 0.98 | 0.25 |
| Leu | CTC | 0 | 12 | 0.00 | 0.21 |
| Met | ATG | 4 | 4 | 1.00 | 1.00 |
| Asn | AAT | 0 | 22 | 0.00 | 0.44 |
| Asn | AAC | 50 | 28 | 1.00 | 0.56 |
| Pro | CCG | 0 | 7 | 0.00 | 0.18 |
| Pro | CCA | 0 | 13 | 0.00 | 0.33 |
| Pro | CCT | 39 | 14 | 1.00 | 0.36 |
| Pro | CCC | 0 | 5 | 0.00 | 0.13 |
| Gln | CAG | 23 | 9 | 1.00 | 0.39 |
| Gln | CAA | 0 | 14 | 0.00 | 0.61 |
| Arg | AGG | 1 | 1 | 0.02 | 0.02 |
| Arg | AGA | 47 | 20 | 0.98 | 0.42 |
| Arg | CGG | 0 | 5 | 0.00 | 0.10 |
| Arg | CGA | 0 | 9 | 0.00 | 0.19 |
| Arg | CGT | 0 | 9 | 0.00 | 0.19 |
| Arg | CGC | 0 | 4 | 0.00 | 0.08 |
| Ser | AGT | 0 | 8 | 0.00 | 0.18 |
| Ser | AGC | 0 | 7 | 0.00 | 0.16 |
| Ser | TCG | 1 | 3 | 0.02 | 0.07 |
| Ser | TCA | 0 | 11 | 0.00 | 0.24 |
| Ser | TCT | 44 | 9 | 0.98 | 0.20 |
| Ser | TCC | 0 | 7 | 0.00 | 0.16 |
| Thr | ACG | 0 | 7 | 0.00 | 0.16 |
| Thr | ACA | 0 | 12 | 0.00 | 0.27 |
| Thr | ACT | 44 | 15 | 1.00 | 0.34 |
| Thr | ACC | 0 | 10 | 0.00 | 0.23 |
| Val | GTG | 0 | 14 | 0.00 | 0.36 |
| Val | GTA | 0 | 2 | 0.00 | 0.05 |
| Val | GTT | 39 | 14 | 1.00 | 0.36 |
| Val | GTC | 0 | 9 | 0.00 | 0.23 |
| Trp | TGG | 10 | 10 | 1.00 | 1.00 |
| Tyr | TAT | 0 | 7 | 0.00 | 0.37 |
| Tyr | TAC | 19 | 12 | 1.00 | 0.63 |
| Stop | TGA | 1 | 1 | 1.00 | 1.00 |
| Stop | TAG | 0 | 0 | 0.00 | 0.00 |
| Stop | TAA | 0 | 0 | 0.00 | 0.00 |
