# Supplementary material for: Minimizing IP issues associated with gene constructs encoding the Bt toxin - a case study
Source: BMC Biotechnol. 2024 Jun 3;24:37. doi: 10.1186/s12896-024-00864-3 (PMC11145813; doi:10.1186/s12896-024-00864-3)
Supplement: Supplementary file 5 — Supplementary Material 5 [file 12896_2024_864_MOESM5_ESM.docx]

***
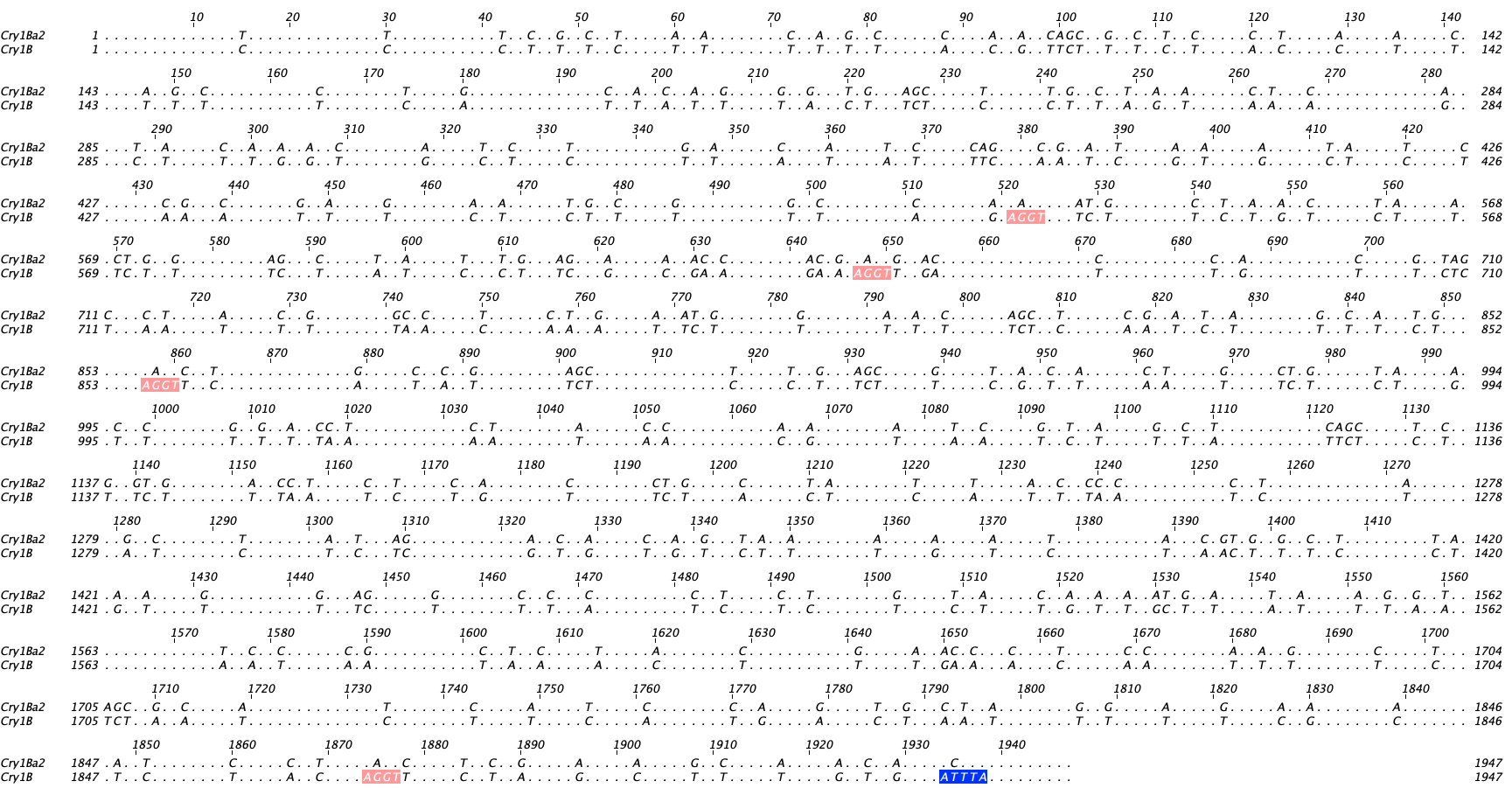
***

**Supplementary Figure 1**. Pairwise alignment of unmodified *Cry1Ba2* (bottom) and the modified *Cry1B^M^* (top) sequences using Jalview [[33]](https://sciwheel.com/work/citation?ids=67944&pre=&suf=&sa=0&dbf=0). Sequences that may function as splice sites in *Cry1B* are shown in red color and the instability motif is shown with blue color. Splice sites are indicated with red color, whereas the ATTTA instability motif is indicated with blue color.
