# Supplementary figures and images for: Minimizing IP issues associated with gene constructs encoding the Bt toxin - a case study
Source: BMC Biotechnol. 2024 Jun 3;24:37. doi: 10.1186/s12896-024-00864-3 (PMC11145813; doi:10.1186/s12896-024-00864-3)

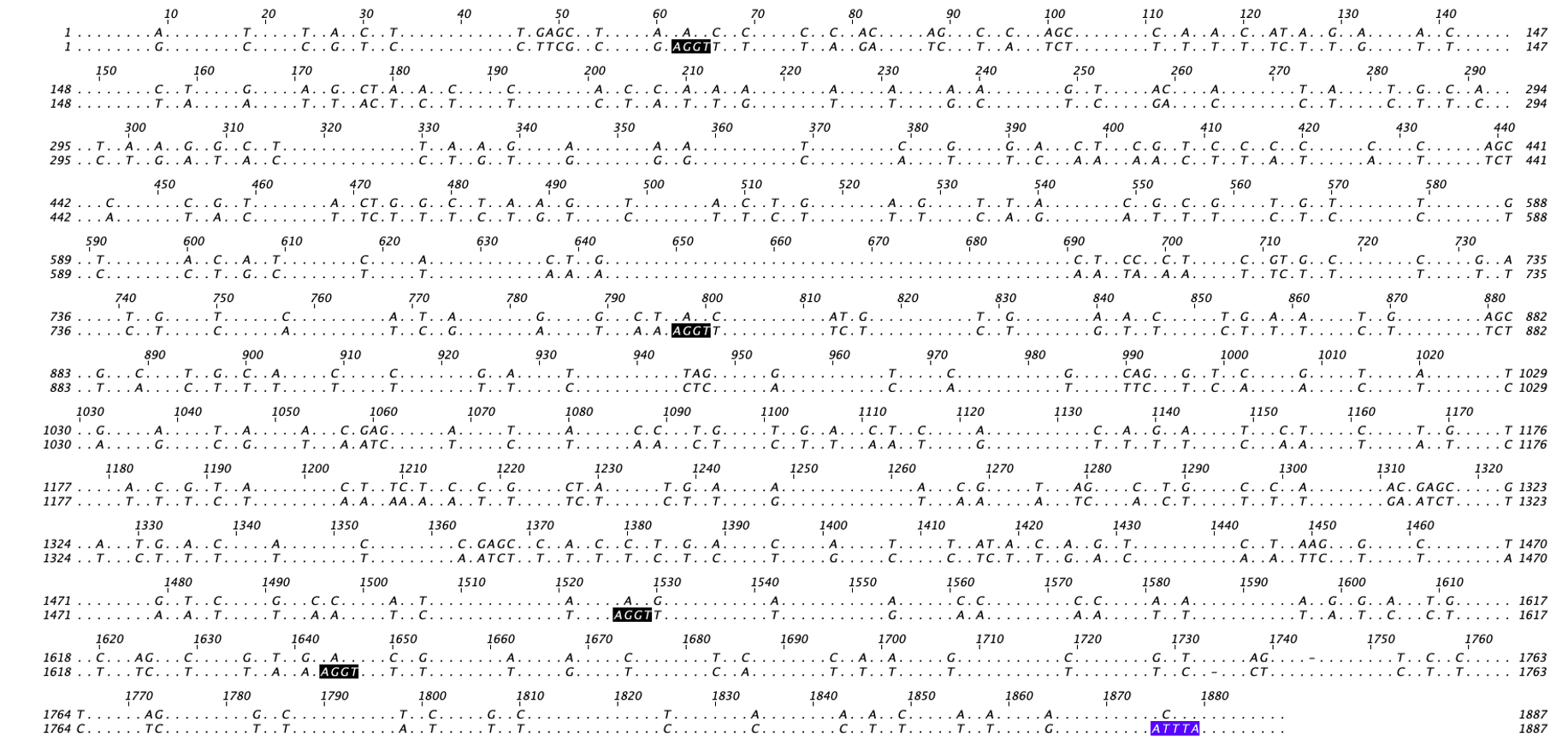

Supplement: Supplementary file 6 — Supplementary Material 6 [file 12896_2024_864_MOESM6_ESM.jpg]

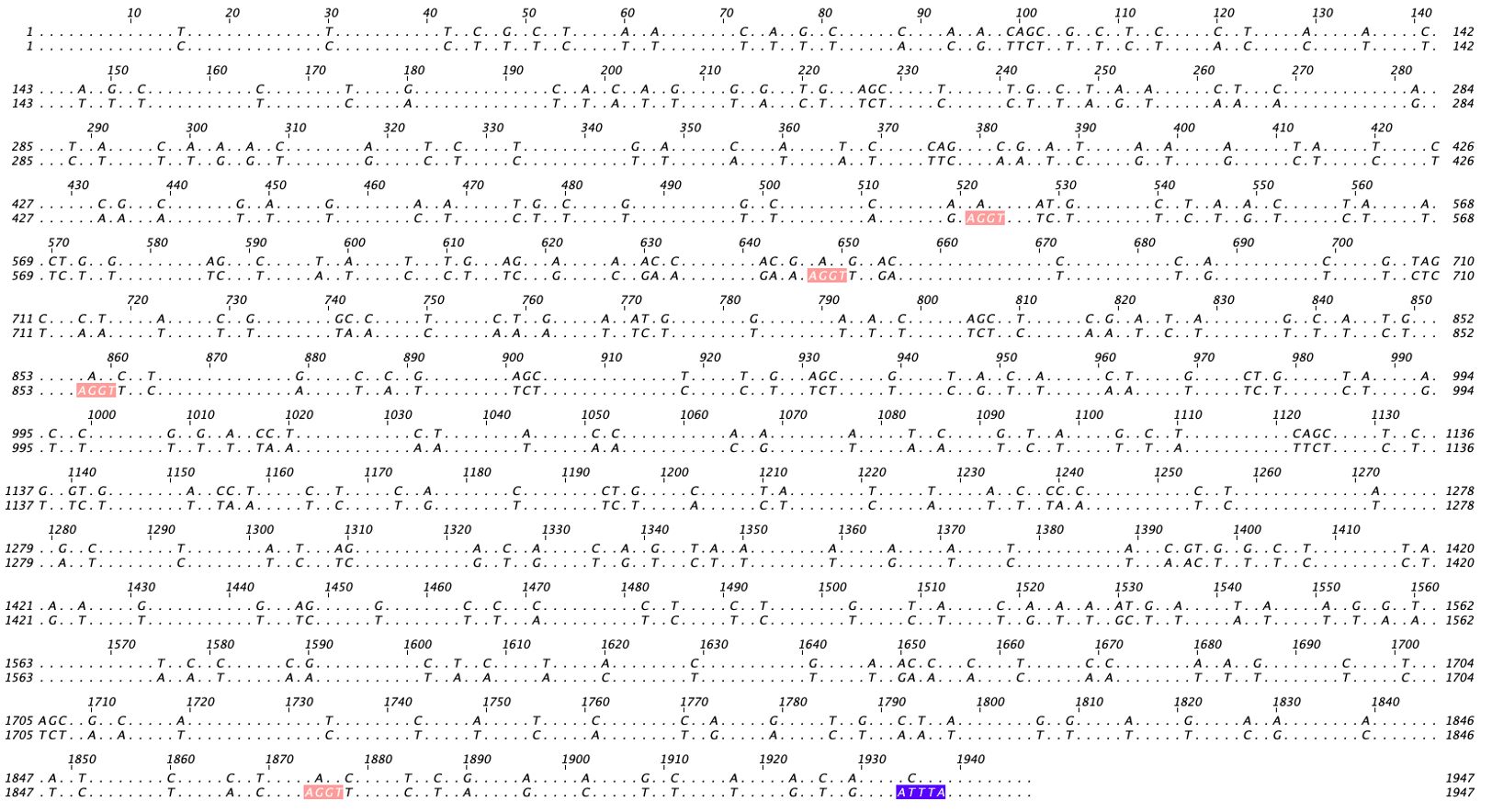

Supplement: Supplementary file 7 — Supplementary Material 7 [file 12896_2024_864_MOESM7_ESM.jpg]
