# Supplementary material for: Minimizing IP issues associated with gene constructs encoding the Bt toxin - a case study
Source: BMC Biotechnol. 2024 Jun 3;24:37. doi: 10.1186/s12896-024-00864-3 (PMC11145813; doi:10.1186/s12896-024-00864-3)
Supplement: Supplementary file 8 — Supplementary Material 8 [file 12896_2024_864_MOESM8_ESM.docx]

**
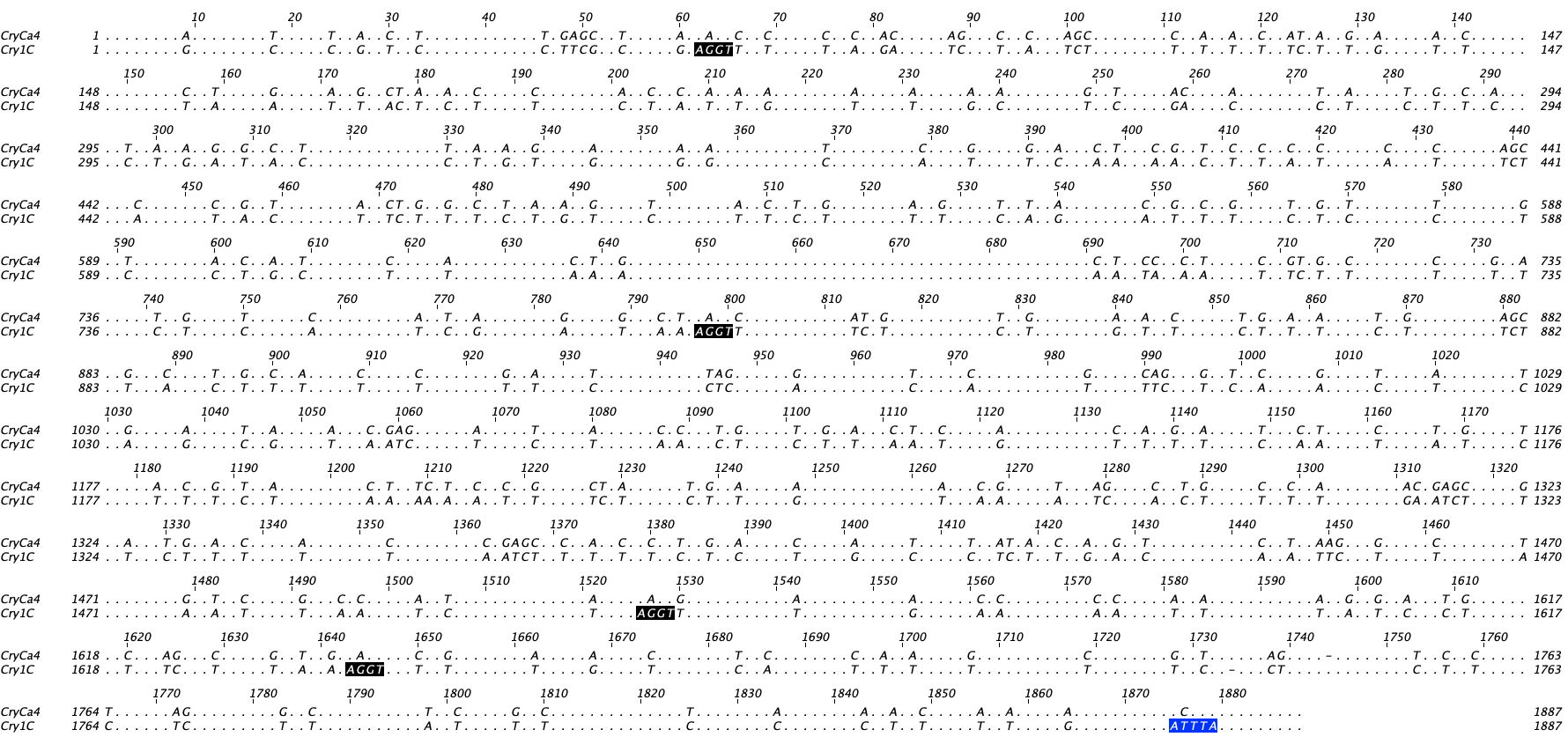
**

**Supplementary Figure 2**: Pairwise alignment of *Cry1Ca4* (bottom) and *Cry1C^M^* (top) sequences. Sequences that may function as splice sites in *Cry1C* are shown in black color and the instability motif is shown with blue color.
